# Evaluation of the Reliability and Quality of YouTube Videos on Congenital Nasolacrimal Duct Obstruction

**DOI:** 10.7759/cureus.36365

**Published:** 2023-03-19

**Authors:** Hasan Burhanettin Kaptı, Burak Erdem

**Affiliations:** 1 Ophtalmology, Ordu University, Ordu, TUR

**Keywords:** youtube, jama score, global quality score, discern score, congenital nasolacrimal duct obstruction

## Abstract

Introduction

Congenital nasolacrimal duct obstruction (CNLDO) causes excessive eye tearing or mucoid discharge. Twenty percent of one-year-olds globally have CNLDO. There are many sources that offer information to parents. This study evaluates the quality and accuracy of CNLDO-related YouTube videos.

Methods

The first 100 videos that appeared after typing "congenital nasolacrimal duct obstruction" in the YouTube search engine were evaluated. These videos were also analyzed and scored using the DISCERN, Journal of the American Medical Association (JAMA), and Global Quality Scoring (GQS) systems.

Results

Forty videos met the inclusion criteria. The mean DISCERN score was 47.3 ± 9.15, JAMA was 1.72 ± 0.87, and GQS was 3.1 ± 0.81. The duration of the videos uploaded by the non-physician group was significantly longer (p = 0.04). In addition, the JAMA score of the videos uploaded by the physician group was significantly higher than that of the other group (p = 0.03). Theoretical videos were longer than surgical videos (p = 0.02). DISCERN, JAMA, and GQS scores were statistically higher in the theoretical video group (p = 0.002, p = 0.04, and p = 0.03, respectively).

Conclusion

According to our research, the quality of YouTube videos about congenital nasolacrimal duct obstruction is average. This information source can be improved by making videos with more detailed information about the disease and theoretical information, as well as by having health professionals look over the content that has been uploaded.

## Introduction

The Internet has made accessing information more convenient. The Internet's enhanced accessibility to information has resulted in a rise in health-related searches. Doctors utilize videos about their respective fields to augment and update their previous knowledge, and people use the Internet to research their health issues. However, the vast majority of the material on the Internet is inaccurate or insufficient. This requires authorities to evaluate the Internet's content.

According to studies conducted in 2019, YouTube is the second-most-viewed website in the world. Due to its user-friendliness and accessibility, it is a platform where individuals may post movies on a range of subjects. There are both good and bad elements to this circumstance. We may claim that the speed of acquiring accurate information from a trustworthy source has grown, but so has the speed and chance of obtaining unreliable and fraudulent information. We know that patients, medical students, physicians, and healthcare assistants routinely use YouTube videos for information [1, 2]. Therefore, understanding the quality and reliability of the received information becomes crucial.

Congenital nasolacrimal duct obstruction (CNLDO) is a common disorder characterized by excessive weeping or mucoid discharge from the eyes as a result of a blocked nasolacrimal duct system. Nasolacrimal duct blockage affects up to 20% of infants under one year of age globally and is commonly reversible without surgery. In addition to invasive therapy, conservative treatment options include observation, lacrimal sac massage, and antibiotics [3].

Parents of babies with CNLDO who are concerned may get information from a variety of sources. In addition, medical students and physicians utilize the Internet to discover how this condition, which is prevalent in children and has various treatment choices, operates. As a result of this, medical professionals who interact with children should exercise caution about the quality and credibility of any medical material they discover online. Previous studies have examined the veracity of YouTube-based information for different pediatric disorders [4-7]. The purpose of this study was to analyze the quality and accuracy of YouTube videos on the management of CNLDO.

## Materials and methods

The study was conducted at the Faculty of Medicine, Ordu University, Ordu, Turkey. Because it was an observational study and used data that was readily available to the public, this study was exempt from ethical review. On December 13, 2022, we analyzed the first 100 YouTube results for the search phrase "congenital nasolacrimal duct obstruction." The search was performed without login, clearing the search history. The default search setting used was "Sort by relevance." Only English videos are included. Videos shorter than thirty seconds, repetitive, unrelated to congenital nasolacrimal duct obstruction, and containing advertisements were excluded. The duration of the videos (minutes), the number of views, the uploader (doctors, health institutions, medical channels), the number of subscribers, the number of likes, the time passed from the upload date (days), and the video's content (surgery or theoretical information) were recorded.

The DISCERN, Journal of the American Medical Association (JAMA), and Global Quality Score (GQS) systems were used to determine the quality and educational value of the videos. DISCERN is a grading system that includes 16 questions and three components to assess the dependability and quality of health information [8]. Each query receives a score between one and five. The first eight questions are used to figure out how trustworthy the page is. In the second part, from questions nine through 15, the quality of the information is evaluated. A broad opinion of the website is asked in question 16. The total score can be anywhere from 16 to 75. A score of 16 to 26 is very bad, 27 to 38 is bad, 39 to 50 is average, 51 to 62 is good, and 63 to 75 is excellent (Table 1).

**Table 1:** The DISCERN scoring system

Question	Score
Section 1: Is the publication reliable?	
Are the aims clear?	1-5
Does it achieve its aims?	1-5
Is it relevant?	1-5
Is it clear what sources of information were used to compile the publication (other than the author or producer)?	1-5
Is it clear when the information used or reported in the publication was produced?	1-5
Is it balanced and unbiased?	1-5
Does it provide details of additional sources of support and information?	1-5
Does it refer to areas of uncertainty?	1-5
Section 2: How good is the quality of information regarding treatment choices?	
Does it describe how each treatment works?	1-5
Does it describe the benefits of each treatment?	1-5
Does it describe the risks of each treatment?	1-5
Does it describe what would happen if no treatment is used?	1-5
Does it describe how the treatment choices affect the overall quality of life?	1-5
Is it clear that there may be more than 1 possible treatment choice?	1-5
Does it provide support for shared decision-making?	1-5
Section 3: Overall rating of the publication	
Based on the answers to all of the above questions, rate the overall quality of the publication as a source of information about treatment choices	1-5

JAMA criteria were used to assess the websites' fundamental information. The JAMA rating system is a method for assessing the credibility of internet health-related content sources. JAMA standards examine the authorship, credit, disclosure, and currency of online medical content [9]. Each criterion is awarded one point, with one point representing the lowest quality and four points representing the best quality (Table 2).

**Table 2:** The Journal of American Medical Association (JAMA) benchmark criteria

Criteria	Description
Authorship	Authors and contributors, their affiliations, and relevant credentials should be provided
Attribution	References and sources for all content should be listed clearly, and all relevant copyright information should be noted
Disclosure	Disclosure of all financing, sponsorship, support, advertising, and video ownership conflicts of interest is required.
Currency	The dates when the content was posted and updated should be included

The GQS system gives people a five-point Likert scale to use to judge the overall quality of a video [10]. GQS gives the chance to comment on the videos as a whole and analyzes the videos' overall quality based on the information flow delivered. The GQS scale spans from one to five (Table 3).

**Table 3:** Global Quality Scoring System

Score	Description
1	Poor quality, and unlikely to be useful to patients.
2	Poor quality, yet some information is present; patients will find it of little use.
3	Suboptimal flow, some information covered but essential issues omitted; patients may find it useful.
4	Good quality and flow, most critical issues covered; beneficial to patients
5	Excellent quality and flow; extremely beneficial to patients

Two experienced ophthalmologists (HBK and BE) gave each video a score on its own, and the mean values of the DISCERN score, JAMA score, and GQS were analyzed statistically.

All statistical analyses of the collected data were performed using the IBM Statistical Package for Social Sciences (SPSS) for Windows version 25.0. The mean ± standard deviation, or mean, was used to present continuous data. Student's t-test for normally distributed continuous variables; Mann-Whitney U test was used for non-normally distributed continuous variables. Spearman's rank correlation analysis was used to evaluate the relationships between the variables. A p-value less than 0.05 was determined to be statistically significant.

## Results

A total of 100 videos were viewed. Analysis was done on 40 videos that fit the requirements for inclusion. The mean DISCERN score of all videos was 47.3 ± 9.15, the mean JAMA score was 1.72 ± 0.87, and the mean GQS was 3.1 ± 0.81 (Table 4). Based on the average DISCERN rating, all videos were of average quality. Based on the DISCERN score, 15% (n = 6) of the videos were bad, 55% (n = 22) moderate, 17.5% (n = 7) were good, and 12.5% (n = 5) were excellent quality.

**Table 4:** Descriptive statistics of YouTube videos Data are shown as mean or mean ± standard deviation; JAMA: Journal of the American Medical Association; GQS: Global Quality Score; Min: minimum; Max: maximum

	Mean ± SD	Min-Max
Length of video (min)	9.9 ± 13.6	2-83
View count	20340 ± 53281	7-292000
Subscribers	38926 ± 119530	26-740000
Likes	144.85 ± 376.69	0-2300
Time since upload(day)	1012.5 ± 842.13	30-3240
DİSCERN score	47.3 ± 9.15	33-68
JAMA score	1.72 ± 0.87	1-4
GQS	3.1 ± 0.81	2-5

Eighteen videos (40%) were posted by medical channels, seven (17.5%) by health institutes, and 15 (37.5%) by physicians. Intraclass correlation (ICC) coefficients were used to evaluate the agreement between masked reviewers who scored DISCERN, JAMA, and GQS, and Spearman's rank correlation coefficient was found to be greater than 0.90.

The videos were categorized as belonging to physicians and non-physicians according to the upload source. Significantly longer videos were submitted by non-physicians than by physicians (p = 0.04). In addition, the JAMA score of the videos uploaded by the physician group was significantly higher than that of the other group (p = 0.03). The two groups did not significantly differ from one another in terms of other viewer interaction parameters, the DISCERN score, and the GQS (Table 5).

**Table 5:** Comparison of videos uploaded by physicians and non-physicians Data are shown as mean or mean ± standard deviation; JAMA: Journal of the American Medical Association; GQS: Global Quality Score

	Physicians (n=15)	Non-physicians (n=25)	p-value
Length of video (min)	5.25 ± 3.34	12.68 ± 16.53	0.04
View count	32590.26 ± 76780.25	12990.92 ± 32003.75	0.13
Subscribers	14179.06 ± 24487.59	53775 ± 149180	0.15
Likes	226.33 ± 583.94	95.96 ± 158.22	0.14
Time since upload (day)	1160 ± 903.3	924 ± 809.11	0.19
DİSCERN score	44.33 ± 5.31	49.08 ± 190.52	0.05
JAMA score	1.92 ± 0.99	1.4 ± 0.5	0.03
GQS	3 ± 0.65	3.16 ± 0.89	0.27

Twenty-seven (67.5%) of the videos included theoretical information, and 13 (32.5%) of them were surgical (Table 6). The mean duration of videos with theoretical information was significantly longer than videos with surgical content (p = 0.02). On the other hand, the DISCERN, GQS, and JAMA scores were statistically significantly higher in the video group containing theoretical information than in the other group (p=0.002, p=0.04, and p=0.03, respectively).

**Table 6:** Comparison of videos with and without surgery Data are shown as mean or mean ± standard deviation; JAMA: Journal of the American Medical Association; GQS: Global Quality Score

	Videos with surgery	Non-surgical videos	P value
Length of video (min)	3.84±2.51	12.81±15.75	0.02
View count	21237.15±30208.1	19909±61939	0.47
Likes	96.3±125.6	168.2±451.4	0.28
Time since upload (day)	1495.38±1005.92	780±651.1	0.004
DİSCERN score	41.53±5.02	50.07±9.45	0.002
JAMA score	1.38±0.5	1.88±0.97	0.04
GQS	2.76±0.43	3.25±0.9	0.03

Relationships between the DISCERN score, JAMA score, GQS, length of videos, number of views, number of subscribers, and number of likes were analyzed (Table 7). The number of views across all videos and the number of likes were significantly correlated (p<0.001; r = 0.951). A positive correlation was observed between the length of the videos and the DISCERN score (r = 0.542, p = 0.001), JAMA score (r4723*-+-0+ = 0.307, p = 0.040), and GQS (r = 0.409, p = 0.001). Also, there was a strong correlation between the DISCERN, GQ6S, and JAMA scores (p 0.001)

**Table 7:** The correlation between some video metrics and the scoring system JAMA: Journal of the American Medical Association; GQS: Global Quality Score

	Length of video (min)	View count	Subscribers	Likes	DİSCERN score	JAMA score	GQS
Length of video (min)	- -	r=-0.126 p=0.200	r=-0.04 p=0.674	r=-0.109 p=0.138	r=0.542 p=0.001	r=0.307 p =0.040	r=0.409 p=0.001
View count	r=-0.126 p=0.200	- -	r=0.081 P=0.561	r=0.951 p<0.001	r=-0.160 p=0.367	r=-0.229 p=0.096	r=-0.076 p=0.543
Subscribers	r=-0.04 p=0.674	r=0.081 p=0.561	- -	r=0.108 p=0.456	r=-0.095 p=0.415	r=-0.036 p=0.602	r=-0.016 p=0.676
Likes	r=-0.109 p=0.138	r=0.951 p<0.001	r=0.108 p=0.456	- -	r=-0.118 p=0.330	r=-0.233 p=0.128	r=-0.070 p=0.564
DİSCERN score	r=0.542 p=0.001	r=-0.160 p=0.367	r=-0.095 p=0.415	r=-0.118 p=0.330	- -	r=0.686 p<0.001	r=0.801 p<0.001
JAMA score	r=0.307 p =0.040	r=-0.229 p=0.096	r=-0.036 p=0.602	r=-0.233 p=0.128	r=0.686 p<0.001	- -	r=0.691 p<0.001
GQS	r=0.409 p=0.001	r=-0.076 p=0.543	r=-0.016 p=0.676	r=-0.070 p=0.564	r=0.801 p<0.001	r=0.691 p<0.001	- -

## Discussion

In our research, we analyzed the quality of YouTube videos concerning CNLDO. Several ophthalmology-related studies have questioned the dependability and quality of YouTube videos. [1,6,7,11]. However, as far as we are aware, this is the first investigation into CNLDO. In this investigation, we did not confine ourselves to a single rating method; rather, we used three distinct scoring systems: DISCERN, JAMA score, and GQS. It was believed that the conclusion would be more objective since each rating system evaluated websites from a unique standpoint.

In our study, all videos were determined to be of low quality based on the mean JAMA score (1.72 ± 0.87), medium quality based on the mean GQS (3.1 ± 0.81), and medium quality based on the mean DISCERN score (47.3 ± 9.15). The mean DISCERN score in a study examining YouTube videos about eyelids was 25.17 ± 6.88, the mean JAMA score was 0.79 ± 0.63, and the average GQS was 2.84 ± 1.03 [12]. It was noted that the videos were of poor quality and were insufficiently educational in a YouTube study on uveitis, which had a mean DISCERN score of 38.5 ± 13.2, a mean JAMA score of 1.8 ± 0.6, and a mean GQS score of 2.5 ± 0.9 [13]. Less than one-third of the 162 YouTube videos analyzed in another study on retinitis pigmentosa were found to be scientifically useful, and 82 out of 162 were misleading [14]. According to Sahin et al., one-third of the YouTube videos about retinopathy of prematurity are false and can have negative effects [11]. The degree of specificity of the research topic, the quantity of videos, and the length of the videos may all have an impact on the outcomes of YouTube studies in the field of ophthalmology.

In a study evaluating eyelid loading videos, a correlation was found between video length and the DISCERN score [12]. In another study on uveitis, a correlation was observed between video length and DISCERN, JAMA, and GQS [13]. Similarly, our study revealed a positive correlation between video length and the DISCERN score, JAMA score, and GQS. It is anticipated that the video will be longer in order to provide a more in-depth explanation of surgical procedures as well as to demonstrate and explain examination, indication, postoperative follow-up, and complications. This is why longer videos may be more educationally beneficial.

Youtube videos were found to be of low quality and inadequate in a study on vitrectomy surgery in vitreous hemorrhages, and the mean DISCERN score was 37.2 ± 6.5, the mean JAMA score was 1.9 ± 0.5, and the mean GQS was 2.0 ± 0.5. The median DISCERN score was 32.8 ± 10, the median JAMA score was 1.3 ± 0.5, and the median GQS score was 3.1 ± 1.1 in a study evaluating YouTube videos on ptosis surgery, and it was emphasized that the videos were not a credible educational information source [15,16]. YouTube videos about keratoconus, on the other hand, were found to be educational and instructive, with a mean DISCERN score of 42.92 ± 18.14, a mean JAMA score of 2.7±0.72, and a mean GQS score of 3.07±1.25 [17]. Studies indicate that YouTube videos with surgical content have a low educational value, and our data support this conclusion. In our study, we compared surgical and non-surgical content videos, and we discovered that the DISCERN score, JAMA score, and GQS score were statistically significantly higher in the non-surgical group than in the surgical group. Using a video to explain the steps and procedure of surgery may be more difficult and complicated than delivering illness information. Moreover, due to the fact that the DISCERN, JAMA, and GQS systems were not intended to assess surgical methods, videos incorporating such techniques may get a lower grade.

According to certain research, the quality and dependability of videos produced by physicians are greater than those uploaded by non-physicians [1, 18]. The DISCERN score and GQS score for videos submitted by physicians and non-physicians were identical, according to our analysis. However, the JAMA score for physicians was statistically significantly higher. In addition, the average duration of the videos uploaded by the non-physician group was statistically significantly higher than that of the physician group. We cannot exclude the possibility that the difference in video durations between the two groups may affect the DISCERN score, JAMA score, and GQS. On the other hand, the high JAMA score of the videos uploaded by physicians reveals that the videos uploaded by physicians are more reliable and have a higher academic value.

Limitations

This research has several limitations. First, we evaluated the videos at a particular moment. YouTube's content may change over time due to the dynamic nature of YouTube's parameters, and different results may be obtained at different times. More studies are needed to analyze the videos in more detail and over longer periods. Additionally, generalizations are impossible because only English-language videos were evaluated. Even though this makes it hard to generalize the study's results, English is known to be the most common language used on the Internet. In our study, we were only able to evaluate 40 videos; this small sample size is insufficient to generalize about internet videos. Nonetheless, we reviewed 40 of the initial 100 videos. This may have resulted in the exclusion of some videos; however, it is doubtful that consumers will see videos beyond the top 100 in search results. YouTube is the most popular place to share videos around the world, but it is not the only place to find videos. Other video-sharing sites like Facebook Watch, Instagram Reels, Vimeo, and TikTok may also have relevant videos, but this study doesn't look at them.

## Conclusions

According to the results of our research, the quality of the videos on YouTube on congenital nasolacrimal duct obstruction is average. The preparation of videos that provide more detailed information about the disease and contain theoretical information, as well as the examination of the uploaded content by health professionals, can increase the quality of this information source.
